# Gold Nanoparticles Modulate Excimer and Exciplex Dynamics of PDDCP-Conjugated Polymers

**DOI:** 10.3390/polym16172420

**Published:** 2024-08-26

**Authors:** Khalid H. Ibnaouf, Ahmed Alsadig, Hajo Idriss, Moez A. Ibrahem, Humberto Cabrera

**Affiliations:** 1 Physics Department, College of Science, Imam Mohammad Ibn Saud Islamic University (IMSIU), Riyadh 13318, Saudi Arabia; hiidriss@imamu.edu.sa (H.I.); maimohammed@imamu.edu.sa (M.A.I.); 2 Institute of Radiopharmaceutical Cancer Research, Helmholtz-Zentrum Dresden-Rossendorf, 01328 Dresden, Germany; a.alsadig@hzdr.de; 3 CNR NANOTEC Institute of Nanotechnology, via Monteroni, 73100 Lecce, Italy; 4 Mlab, STI Unit, The Abdus Salam International Centre for Theoretical Physics, 34151 Trieste, Italy; hcabrera@ictp.it

**Keywords:** conjugated polymer, gold nanoparticles, exciplex, LIF, ASE

## Abstract

How plasmonic nanostructures modulate the behavior of exciplexes and excimers within materials remains unclear. Thus, advanced knowledge is essential to bridge this gap for the development of optoelectronic devices that leverage the interplay between plasmonic and conjugated polymer hybrid materials. Herein, this work aims to explore the role of gold nanoparticles (AuNPs) in modulating exciplex and excimer states within the conjugated polymer poly(2,5-di(3,7-dimethyloctyloxy) cyanoterephthalylidene) (PDDCP), known for its photoluminescent and semi-conductive properties, aiming to create innovative composite materials with tailored optical features. The spectral analysis was conducted to investigate the effects of AuNPs on the PDDCP, varying AuNP volume percentages to measure changes in the absorption profile, molar extinction coefficient (*ε*), absorption cross-section (*σ_a_*), and optical bandgap ($E_{g}$). Fluorescence spectra of the mixture at different volume ratios were also examined to assess exciplex formation, while amplified spontaneous emission (ASE) profiles were analyzed to study the behavior and photochemical stability of the polymer–NP hybrid material. Increasing AuNP volume induced both blue and red shifts in the absorption profile of the PDDCP. Higher AuNPs concentrations correlated with decreased *ε* and *σ_a_*, inversely impacting $E_{g}$. The emission spectra obtained at varied AuNP volume ratios indicated significantly enhanced exciplex and excimer formations. The ASE profiles remained consistent but showed reduced intensity with increasing AuNPs concentrations, indicating their influence on hybrid material behavior and stability. The findings suggest that AuNPs affect PDDCP’s optical characteristics, altering the absorption profile, bandgap, and fluorescence spectra. Furthermore, the observed reduction in ASE intensity highlights their influence on the behavior and photochemical stability of the hybrid material. These results contribute to a better understanding of the versatile applications of plasmonic-conjugated hybrid polymers.

## 1. Introduction

A tremendous advancement has been observed over the past few years in the development of nanomaterials, sparking interest in the modification of the properties of the materials, particularly at the nanoscale. Among the various nanomaterials, gold nanoparticles (AuNPs) have received attention from researchers in the field of science and engineering because of their plasmonic abilities and substantial influence on adjacent molecular environments [1,2,3,4,5]. Collective oscillation in conduction band (CB) electrons of nanoparticles in response to incident light of a specific wavelength enhances light-matter interactions due to plasmonic effects [6,7]. Based on the geometry, size, and composition control of the AuNPs, a wide range of optical phenomena have been harnessed, such as fine-tuning of localized surface plasmon resonance (LSPR), scattering, and absorption profiles [8]. The ability to modify the optical properties of a substance using AuNPs has been an important factor in various scientific and technological fields, including photonics [9,10,11], optoelectronics [10,12,13,14], sensing [15], bioimaging [16], and catalysis [17]. The synergistic coupling not only advanced these areas but also boosted capacity in light harvesting and the development of more innovative composites with different optical characteristics. The process of integrating plasmonic nanoparticles with conjugated polymers (CPs) has shown some promise in the development of innovative devices with desirable properties for optoelectronic and catalytic applications [18,19]. Conjugated polymers are a distinct class of organic materials that possess remarkable optical features. They have drawn researchers’ attention due to their versatile applications [20,21,22]. They are differentiated by their comb-like architectural design consisting of carbon frameworks with characteristic single and double bonds as well as flexible alkyl side chains [23,24]. Their unique characteristic of alternating bonds in the carbon backbone results in delocalized π-electrons, which enable effective light absorption and ease of charge transport. This allows the materials to be utilized across different fields of optoelectronic applications [25,26,27]. In this context, we previously investigated the spectral properties of the CP, referred to as 9,9-dioctylfluorenyl-2,7-diyl (PFO), in various solutions and across different temperatures [28]. The results showed that PFO tended to aggregate in the liquid state, driven by CH-π interactions between molecules in the ground state. This interaction was evident in the absorption spectra, where a new band appeared at 437 nm at higher concentrations and lower temperatures. In another work, we studied the photophysical properties of the conjugated polymer poly [2-methoxy-5-(3′,7′-dimethyloctyloxy)] (MDMO-PPV) across a range of solvents and concentrations. The absorption spectrum showed two bands at 333 and 490 nm. The fluorescence spectrum exhibited two bands at 555 and 595 nm. Unlike other PPV derivatives, the intensity ratio of these bands was unaffected by concentration or temperature. These bands (555 and 595 nm) are attributed to the vibronic transitions (0–0 and 0–1, respectively) [29]. The intermolecular interactions within the conjugated polymers give rise to important optical attributes, such as exciplexes and excimers. The term exciplex refers to transient complex due to two dissimilar molecules, each having different electronic properties in an excited state, resulting in the efficient transfer of energy and the creation of new excitation upon emission [30]. An excimer refers to the excited-state dimers that result when two identical molecules appear together in an excited-state, forming a red-shifted emission in comparison to each of the molecules [31]. The combination of CPs with metal nanoparticles (MNPs), especially AuNPs, provides a platform for exploring novel exciplex formation. The spatial arrangement and electronic coupling between the CPs and the MNPs are vital for the formation of the exciplex. The CPs acted as donors of electron during exciplex formation, particularly after the photons are absorbed and become excited [32]. On the other hand, when in proximity to the CPs, the MNPs accept the electron. The plasmonic properties of the MNPs facilitate efficient energy transfer and promote the exciplex formation. As such, the LSPR enhances energy transfer processes from the CPs to the MNPs and forms a highly stable exciplex [33,34,35]. Thus, researchers have reported on exciplex formation by coupling various CPs with MNPs for various applications. For instance, co-polymers of poly(9,9-dioctylfluorene-co-3,4-ethylenedioxythiophene) otherwise called (PDOF-co-PEDOT) were blended with silver nanoparticles (AgNPs) and employed as sensors for pesticide detection [36]. AgNPs promote the surface-enhanced Raman scattering (SERS) effect of the exciplex molecule, improving its sensitivity for the detection of the pesticides [36]. In another study, the plasmonic effect for the interaction of N,N′-di(1-naphthyl)-N,N′-diphenyl-(1,1′-biphenyl)-4,4′-diamine (NPB) and carbazole derivative 4,4′-bis(N-carbazolyl)-1,1′-biphenyl (CBP) with aluminum nanoparticle (Al-NP) arrays was also reported [6]. Al-NP incorporation using a e-beam lithography technique into the NPB/CBP has been shown to improve the efficiency of the exciplex light emission diode [37]. More recently, we reported the potential transformative effect of zinc oxide nanoparticles (ZnO NPs) on the spectral properties of MDMO-PPV polymer based on different ratios of ZnO NPs concentrations on glass substrates. Adding 5% ZnO NPs led to a dramatic alteration in the UV–Vis spectrum. A significant reduction in the absorption at the 490 nm band was observed, while the absorption at 333 nm increased rapidly and became more pronounced. When the ZnO NP concentration was increased to 10%, no noticeable change occurred in the 490 nm band; however, the 333 nm band shifted towards the blue region [38].

Although the impacts of nanostructures with distinct plasmonic optical features on the CPs have been reported [39,40,41,42,43], a deep understanding of how AuNPs the behaviors of exciplexes and excimers within the materials is lacking. Thus, advance knowledge is needed to bridge the gap for the optoelectronic devices that capitalize on the interplay between plasmonic CPs and hybrid materials. Moreover, a better understanding of the performance of plasmonic CPs in liquid form could provide more insights into their behaviors as well as potential applications. In this work, poly(2,5-di(3,7-dimethyloctyloxy) cyanoterephthalylidene (PDDCP) was employed. We previously reported the impact of the concentration of this polymer dissolved in tetrahydrofuran (THF) on its optical characteristics [44]. The result showed significant modifications in the photoluminescence spectrum (PL), which might be due to the exciplex and excimer formations. Furthermore, it was observed that the fluctuations in the energy band gap were dependent on the concentration. The current work is aimed at analyzing the amplified spontaneous emission (ASE) properties, optical profile, and photochemical stability of PDDCP doped with different quantities of AuNPs. On the other hand, the energy band gap, refractive index, and absorption cross-section were computed.

## 2. Materials and Methods

### 2.1. Synthesis of Colloidal AuNPs

Gold colloid was prepared through the reduction of chloroauric acid (HAuCl_4_) using the sodium citrate reduction approach as reported in [15]. All glassware was cleaned and left to dry overnight before the synthesis process. Here, 1.1 mL of 17.3 mM HAuCl_4_ was introduced to Milli-Q water (43 mL), which was continuously stirred and boiled (100 °C). Then, 300 μL of 245 mM trisodium citrate was promptly added, and the solution was stirred for 30 min for the complete reduction of the gold salt (HAuCl_4_). All the chemical reagents were purchased from Sigma-Aldrich (St. Louis, MO, USA). The colloids of AuNPs formed were kept at room temperature and protected from exposure to light prior to use. The AuNPs formed were characterized using UV–Vis spectrophotometry, transmission electron microscopy (JEOL JEM 2010F, JEOL Ltd., Tokyo, Japan), and dynamic light scattering.

### 2.2. Preparation of Polymer (PDDCP) Doped AuNPs

**Figure 1:**
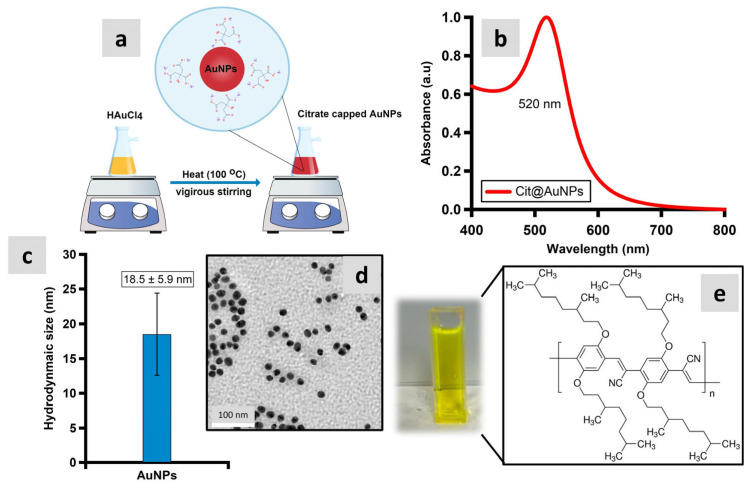
(**a**) The Turkevich method for the synthesis of AuNPs. (**b**) The reaction was completed in 30 min, yielding a dispersion of colloidal gold of about 20 nm size, as confirmed by the UV–Vis spectroscopic profile. (**c**) Volume-weighted DLS measurement of the particles. (**d**) A TEM micrograph of AuNPs. (**e**) The PDDCP structure obtained from the datasheet of the manufacturer.

The polymer PDDCP was also obtained from Sigma-Aldrich and used without further treatment. The polymer structure is depicted in Figure 1e. Here, 10 mL of 25 µg/mL (*v*/*v*) of the PDDCP in THF (purity 99.8%, Honeywell Fluka, NJ, USA) was prepared. Then, in a quartz cuvette, 3 mL of various proportions of AuNPs [33, 50, and 75% (*v*/*v*)] were prepared. This process yielded a homogenous solution, denoted as (PDDCP)@AuNPs, which was then examined for its optical characteristics.

### 2.3. Characterization of Polymer PDDCP @AuNPs

**Figure 2:**
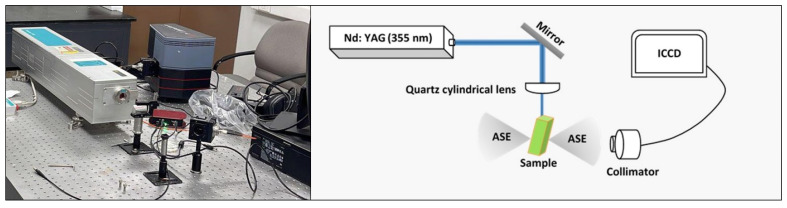
The experimental laser setup used in the study. The illustration provides an overview of the optical device and its corresponding components.

The recorded data of the PDDCP@AuNPs at different concentrations of AuNPs was subjected to absorption and fluorescence spectroscopy. The spectrophotometer (JASCO V-770, Tokyo, Japan) and spectrofluorometer (PerkinElmer LS45, Buckinghamshire, UK) spectra of PDDCP were recorded at room temperature. For amplified spontaneous emission (ASE) spectra, a Nd:YAG laser (Beamtech Nimma-900, Beijing, China) was used as an excitation source at maximum adsorption (*λ_ex_*) of 355 nm. A cylindrical lens made of quartz (f = 5 cm) was focused on the laser beam to perform transverse excitation. By optimizing the pump power and (PDDCP)@AuNPs, the ASE beam of a cone light was generated. The ASE was obtained with the optical fiber, which was pointed into the spectrometer slit (1-mm). The spectral characteristics of the ASE were recorded with a camera (CCD IsoPlane, Princeton Instruments Inc., Trenton, NJ, USA). The experimental setup is depicted in Figure 2.

## 3. Results and Discussion

### 3.1. Characterization of Synthesized AuNPs

Typically, MNPs in solution are attracted to one another by strong van der Waals forces at close range. By adsorbing citrate molecules onto AuNPs, negatively charged ions cover the surface of the nanoparticles, allowing electrostatic repulsion to keep the particles apart. The band of the AuNP surface plasmon resonance (SPR) is shown in Figure 1b. The graph depicts a peak at 520 nm, which aligns to the band of AuNP SPR. The hydrodynamic size of AuNPs was determined using DLS. The calculated average particle diameter was found to be 18.5 ± 5.9 nm, as shown in Figure 1c. Additionally, the extinction coefficient for AuNPs 15–20 nm in diameter was determined to be 3.67 × 10^8^ M^−1^ cm^−1^. Based on this information, the concentration of AuNPs with an approximate diameter of 20 nm was estimated to be 2.06 nM. Further, the morphology of AuNPs were analyzed using TEM microscopy, and the images showed well-defined spherical AuNPs with an average size of 18.5 ± 5.9 nm as shown in Figure 1d.

### 3.2. Spectral Behavior of PDDCP@AuNPs

**Figure 3:**
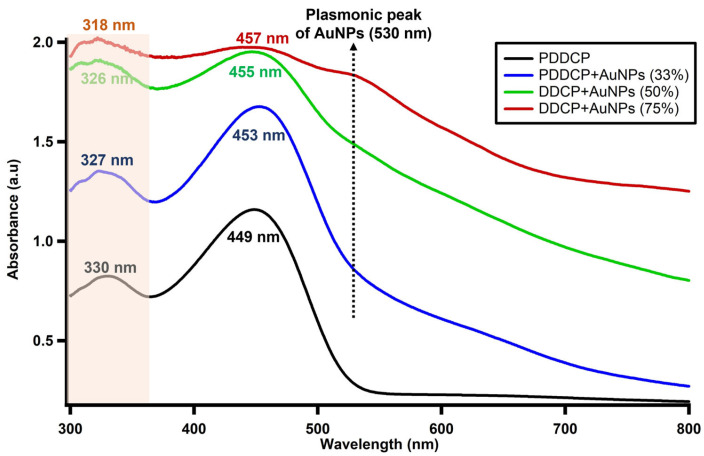
Absorbance of the PDDCP in THF at different AuNP concentrations.

The absorption spectrum of pristine PDDCP in an organic solvent of THF was evaluated at 25 μg/mL. Two absorption bands at 330 and 449 nm are shown (Figure 3). Consequently, these bands correlate with the π-π* electronic transition. Based on our earlier communication, the PDDCP in THF does not exhibit a dimer aggregation phase at high concentrations [44]. To investigate the impact of AuNPs on spectral properties, a homogeneous solution was prepared by adding AuNPs at a volume ratio of 33% (*v*/*v*) to the pure PDDCP at a concentration of 25 μg/mL. The findings displayed a marginal blue shift at a short wavelength (327 nm), while a small red shift occurred in the long wavelength (453 nm). At a volume ratio of 50% AuNPs, the blue and red shifts of the two bands exhibited a rise, resulting in wavelengths of 326 and 455 nm, respectively. Notably, as the volume percentage of AuNPs increased to 75%, the spectral wavelengths (SW and LW) experienced a shift and were thereafter centered at 318 and 457 nm, respectively. It is worth mentioning that increasing the concentration of AuNPs within the polymer enhances the intensity of the absorption band at 327 nm due to the overall structural changes in the hybrid material. The enhanced intensity of this band can be attributed to the charge mobility of the acquired chains, which directly contributes to an increase in the absorption cross-section (*σ*_*a*1_) of the shorter band, as noted in Table 1. Also, an additional band at a wavelength of 520 nm was detected. This band could be ascribed to the plasmonic red shift phenomenon, which can be explained by the existence of polymer layers on the gold surface and the associated polymer binding mechanisms. The Beer–Lambert law and absorption spectrum were employed to compute the extinction coefficient (*ε*) (Equation (1)). The value of *ε* was determined to be on the order of $10^{4}$, as listed in Table 1. This observation strongly suggests that the observed transition can be ascribed to the singlet transition, known as the π-π* transition. Furthermore, the absorption cross-section ${(\sigma }_{a})$ was calculated using Formula (2), which is commonly used to calculate the absorption cross-section in molecular systems [45,46]. A higher value of $\sigma_{a}$ is desirable for a good laser medium.

The findings indicate that there is a linear relationship between both *ε* and $\sigma_{a}$ and the concentration, as demonstrated in Table 1.

(1) $$\varepsilon =\frac{A}{C\times l}$$

In this context, the symbols *ε*, *A*, *C*, and l indicate the extinction coefficient, absorbance, concentration, and length of the cuvette, respectively. The absorption cross-section $\sigma_{a}$ is calculated according to following formula.

(2) $$\sigma_{a}=0.385\times 10^{-20}\times \left(\varepsilon \right)$$

### 3.3. Energy Band Gap ($E_{g}$)

In this work, the energy band gap $\left(E_{g}\right)$ for pure polymer PDDCP and PDDCP@AuNPs determination was carried out using the Tauc method, which is given in Equation (3).

(3) $$\alpha h\nu =B{\left(h\nu -E_{g}\right)}^{n}$$

**Figure 4:**
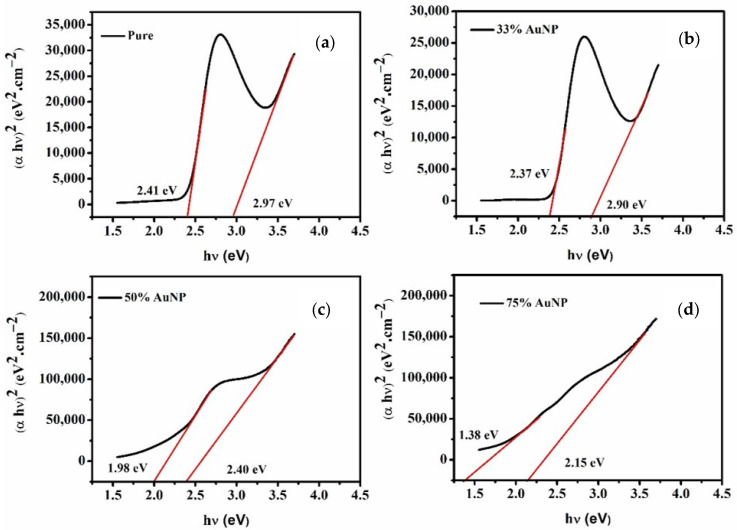
The (*αhv*)^2^ against photon energy of (**a**) pristine and PDDCP mixed with (**b**) 33%, (**c**) 50%, and (**d**) 75% AuNPs.

The hν, α, $E_{g}$, and B depicts the energy of photon, coefficient bandgap of absorption, and band tailing parameter. Here, n=2 for a direct bandgap. For the $E_{g}$ quantification, the curved and straight parts have been extrapolated for the ${\left(\alpha h\nu \right)}^{2}$ and hν as highlighted in Figure 4. The finding signifies that purified polymer PDDCP and PDDCP@AuNPs have two band gaps. It indicates that an increase in AuNPs quantities led to a decrease in $E_{g}$. The observed phenomenon might be attributed to the shift of both the valence and conduction bands. Likewise, the improvement of carrier interactions arises from the elevated charge densities in the VB and CB, resulting in the $E_{g}$ reduction. In addition, the existence of unsaturated defects boosted the density of localized states within $E_{g}$, subsequently leading to a reduction in the optical bandgap. The refractive index (n) as a function of $E_{g}$ was computed using the modified Moss relation [47,48]. The corrected version is shown in Equation (4), and the values are shown in Table 2.

(4) $$n^{4}=\frac{k}{E_{g}}$$

Here, k is a constant = 108 eV. The refractive index values are presented in Table 2.

**Table 2:** The Optical characteristics of Pristine PDDCP and PDDCP@AuNPs.

Samples	${Low\;E}_{g}$ (eV)	${High\;E}_{g}$ (eV)	Refractive Index	
			$n_{1}$	$n_{2}$
PDDCP	2.41	2.97	2.59	2.46
PDDCP@AuNPs (33%)	2.37	2.90	2.60	2.47
PDDCP@AuNPs (50%)	1.98	2.40	2.72	2.59
PDDCP@AuNPs (75%)	1.38	2.15	2.97	2.66

### 3.4. Fluorescence Spectra of PDDCP@AuNPs

**Figure 5:**
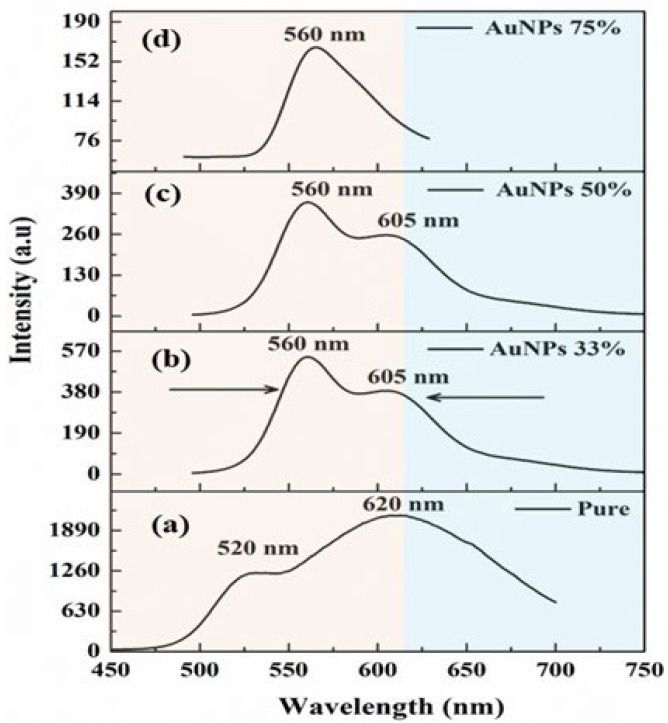
(**a**) The emission spectra of PDDCP in a THF solution at a fixed concentration. The spectral characteristics of the mixture for varying proportions of AuNPs: (**b**) 33%, (**c**) 50%, and (**d**) 75%.

Figure 5a depicts the emission spectrum of PDDCP in THF at 25 μg/mL employing a specific excitation wavelength of 355 nm. Under these operational conditions, the emission spectrum displayed two distinct peaks at 520 nm (shoulder) and 620 nm (dominant) (Figure 5a). The 520 nm band is probably related to dipole–dipole interactions between species of PDDCP and THF, namely in the exciplex state. In contrast, the 620 nm band plausibly results from the interaction between two PDDCP species, with one in the excited state and the other in the ground state, referred to as the excimer state, as demonstrated in our prior work [44]. Adding AuNPs to PDDCP (25 μg/mL) at a volumetric ratio of 33% significantly alters the spectral characteristics of the fluorescence spectrum. Specifically, the shoulder bands at 520 nm became more pronounced and exhibited a red shift towards 560 nm. Conversely, the prevailing band at 620 nm was transformed into a hump band and underwent a blue shift to 605 nm, as seen in Figure 5b. Similarly, no change in the fluorescence spectrum occurs when AuNPs with a volumetric ratio of 50% are introduced (Figure 5c). However, upon increasing the volumetric ratio of AuNPs to 75%, the hump band at 620 nm disappeared, leaving only a single band at 560 nm, with no further shifts observed (see Figure 5d). The inclusion of AuNPs within the polymer strongly supports the hypothesis that the AuNPs play a significant role in enhancing exciplex formation. Furthermore, the incorporation of AuNPs into the polymer reduced the intensity of its emission. The exponential decrease in emission intensity is attributed to the presence of a donor–acceptor contact between AuNPs and polymer molecules. It is well established that metallic surfaces induce strong quenching of molecular fluorescence due to energy transfer or electron transfer from the fluorescent molecules to the metals and the large total surface areas of the nanoparticles. In chromophore/AuNP nanocomposites, the molecular excitation energy of the chromophores can be transferred efficiently to AuNPs, thereby quenching the emission [49,50].

### 3.5. ASE and LIF Properties

**Figure 6:**
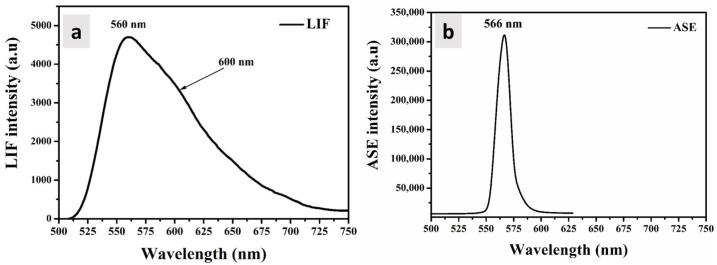
(**a**) The LIF spectrum of PDDCP in 25 µg/mL THF. (**b**) ASE spectrum recorded at a higher polymer concentration of 100 µg/mL.

A pulsed laser excitation of Nd: YAG laser (355 nm) was used with a pump pulse energy of 3 mJ and a pulse repetition of 10 Hz. At the 25 μg/mL concentration, a laser induced fluorescence (LIF) spectrum was obtained that featured two distinct bands centered around 560 and 600 nm (FWHM = 90 nm), as demonstrated in Figure 6a. Here, 3 mJ of energy can cause a population inversion for laser generation, but the ASE was produced at an insufficient concentration. At 100 μg/mL and under identical conditions (including wavelength excitation, pump pulse energy, pulse duration, and temperature), an ASE with a 566 nm peak (10 nm FWHM) was obtained. This ASE peak suggests the potential involvement of an exciplex state, indicating a probable association with exciplex formation (see Figure 6b). Consequently, it becomes apparent that this polymer exhibits a high propensity to remain in its exciplex state when subjected to high concentrations and pumping power energy.

**Figure 7:**
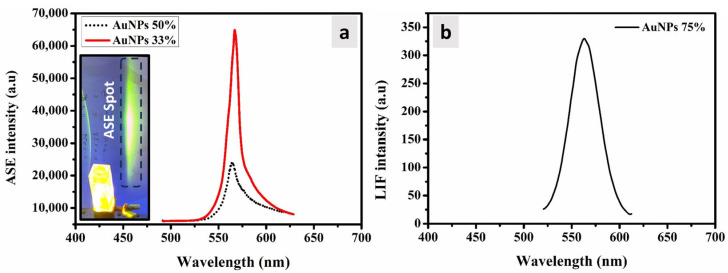
(**a**) ASE spectrum of PDDCP with 33% and 50% AuNPs. Inset: Digital photograph of the ASE emission. (**b**) LIF spectra of the PDDCP and 75% AuNPs.

Having discussed the ASE characteristics of the pristine polymer of PDDCP, the impact of AuNPs on the properties of ASE were identified. Initially, we introduced AuNPs at a volume ratio of 33% into the pure polymer solution (100 μg/mL). Subsequently, we excited the mixture using an Nd: YAG laser. Our observations revealed that the ASE profile remained consistent across various intensities, although it experienced a noticeable reduction in intensity. Simultaneously, the FWHM increased slightly to 12 nm, as observed in Figure 7a. To further investigate this effect, we conducted another experiment by increasing the volume percentage of AuNPs to 50%. The findings indicated that the spectral shape remained unchanged, exhibiting a single peak at 565 nm (Figure 7a). However, the FWHM increased to 17 nm. Consequently, this spectrum can be categorized as laser-LIF, characterized by an FWHM greater than or equal to 15 nm, rather than an ASE spectrum, which typically has an FWHM of 12 nm or less. Using the same experimental conditions, the experiment was repeated with a 75% volume of AuNPs. In this case, only fluorescence emission was detected (Figure 7b), while the super-radiant ASE spot (observed in Figure 7a) had completely vanished. The disappearance of the ASE spectrum can be assigned to the abundance of gold crystals, which scattered the luminous exciting laser source of Nd: YAG laser, leading to the quenching of the ASE spectrum.

### 3.6. Photochemical Stability of the PDDCP@AuNPs

**Figure 8:**
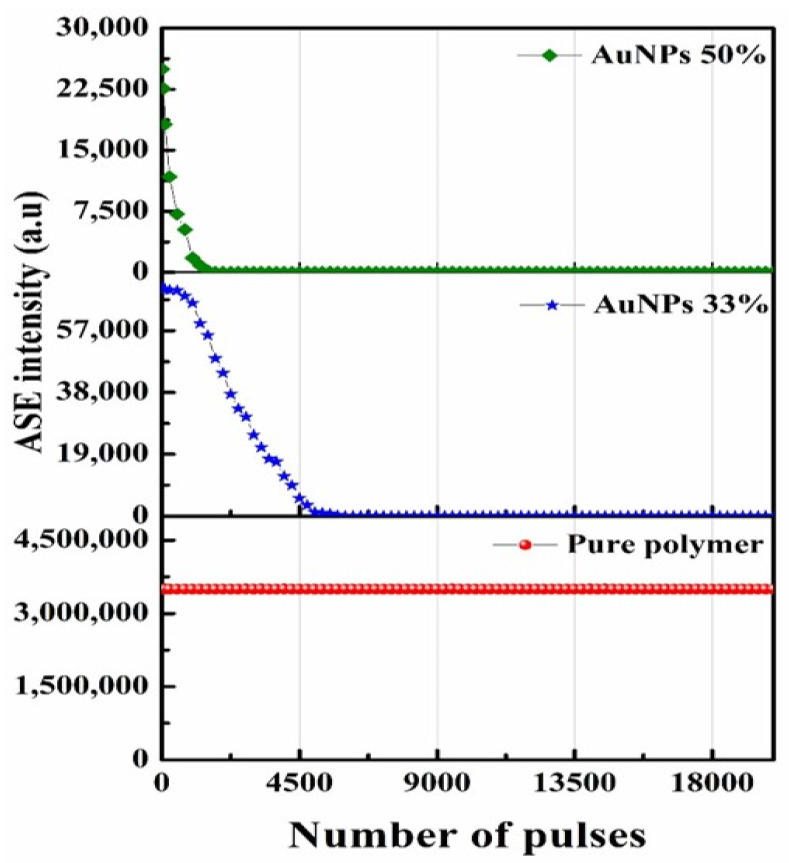
Photochemical stability of the polymer with various AuNP concentrations as a function of the laser pulse.

Next, we devised an experiment to assess the photochemical stability of the polymer and PDDCP@AuNP mixture. We prepared a range of solutions, including pure polymer, a 33% AuNP mixture, and a 50% AuNP mixture. These solutions were subjected to the Nd: YAG laser (355 nm, 3 mJ, and 10 Hz). The stability of the ASE intensity of the pure polymer was found to be remarkably consistent. In contrast, the ASE intensity of the 33% AuNP mixture solution experienced a reduction to 50% of its initial intensity after 2250 pulses, as depicted in Figure 8. Under the same experimental conditions, the ASE intensity of the 50% AuNP mixture vanished completely after 2250 pulses. Unsurprisingly, the PDDCP@AuNPs (75%) did not display any ASE. The plausible explanation is that at higher concentrations of AuNPs, potential aggregation could alter the local environment around the PDDCP polymer. These changes could impact the photophysical properties of the complex, including the ASE profile. Taken together, these findings highlight the significant role played by AuNPs in both the ASE behavior and the photochemical stability of the system.

## 4. Conclusions

The investigation into hybrid plasmonic-conjugated polymer materials in a liquid state offers valuable insights into their interactions and potential applications. The role of spherical AuNPs on the formation of exciplex and excimer states within a selected conjugated polymer referred to as PDDCP was studied. Upon analyzing the spectral behavior of the PDDCP@AuNPs, it was observed that the increase in the volume percentage of AuNPs within the polymer mixture resulted in both blue and red shifts in the absorption profile due the structural changes and the charge mobility in the hybrid material. In addition, as the concentration of AuNPs increased, the molar extinction coefficient (ε) exhibited a linear increase in the absorption cross-section ($\sigma_{a1}$) for the shorter wavelength, while a decrease in ($\sigma_{a2}$) was noted for the longer wavelength. Interestingly, the optical bandgap ($E_{g}$) showed an inverse relationship with the concentration of AuNPs, with values ranging from 2.41 eV for the pure polymer to 2.37 eV, 1.98 eV, and 1.38 eV for mixtures containing 33%, 50%, and 75% AuNPs, respectively. The fluorescence spectra demonstrated that the presence of AuNPs at various volume ratios significantly enhanced exciplex formation. It was also noted that the ASE profile remained consistent across different concentrations but showed an increase in the FWHM as well as a reduction in the intensity. Notably, the PDDCP polymer displayed exceptional photochemical stability compared to the PDDCP@AuNP hybrid material. The substantial reduction in the ASE intensity in solutions containing AuNPs highlights the profound impact on the ASE behavior and the overall photochemical stability of the hybrid material.

## Figures and Tables

**Table 1:** Optical features of PDDCP for THF at different AuNP concentrations.

Volume Ratio of AuNPs (%)	Absorption Band ($\lambda_{1}$) (nm)	Absorption Band ($\lambda_{2}$) (nm)	Extinction $\mathrm{Coefficient}\;(\varepsilon_{2})$ $\;{\left(\frac{g}{100mL}\right)}^{-1}.cm^{-1}$	Absorption Cross $\mathrm{Section}\;(\sigma_{a1})$ $cm^{2}$	Absorption Cross $\mathrm{Section}\;(\sigma_{a2})$ $cm^{2}$
0	330	449	$1.30\times 10^{4}$	3.34 × $10^{-17}$	4.94 × $10^{-17}$
33	327	453	$1.28\times 10^{4}$	4.18 × $10^{-17}$	4.86 × $10^{-17}$
50	326	455	$1.20\times 10^{4}$	4.60 × $10^{-17}$	4.56 × $10^{-17}$
75	318	457	$1.11\times 10^{4}$	4.70 × $10^{-17}$	4.23× $10^{-17}$

## Data Availability

The original contributions presented in the study are included in the article; further inquiries can be directed to the corresponding author.
